# HPV Vaccine Communication Competency Scale for Medical Trainees: Interdisciplinary Development Study

**DOI:** 10.2196/38164

**Published:** 2022-11-04

**Authors:** Gabrielle Darville-Sanders, Humberto Reinoso, Jann MacInnes, Emilie Corluyan, Dominique Munroe, Mary W Mathis, Suzie Lamarca Madden, Johnathan Hamrick, Lisa Dickerson, Cheryl Gaddis

**Affiliations:** 1 Department of Public Health Mercer University Atlanta, GA United States; 2 Georgia Baptist College of Nursing Mercer University Atlanta, GA United States; 3 Department of Research and Evaluation Methodology College of Education The University of Florida Gainesville, FL United States; 4 Department of Public Health Mercer University Macon, GA United States; 5 College of Pharmacy Mercer University Atlanta, GA United States; 6 Physician Assistant Studies Mercer University Atlanta, GA United States

**Keywords:** human papillomavirus, HPV, HPV vaccine, provider communication, medical trainees, immunization, vaccine, communication, student, sexually transmitted infection, STI, United States of America, USA, young adult, teen, patient, parent, immunization, mobile phone

## Abstract

**Background:**

Human papillomavirus (HPV) infection is the most common sexually transmitted infection in the United States. High-risk HPV strains are associated with cancer of the cervix, oropharynx, anus, rectum, penis, vagina, and vulva. To combat increasing HPV-related cancers, the 9-valent HPV vaccine Gardasil was developed. Recommendation of the HPV vaccine by a health care provider has been cited as the number one factor affecting vaccine uptake among adolescents and young adults. Physician assistants, nurse practitioners, and pharmacists have been enlisted to bridge the gap.

**Objective:**

The specific aim of this research study was to develop a reliable and valid HPV vaccine communication scale that can be used to measure the competency of primary care providers when recommending the need for vaccination to parents and patients.

**Methods:**

Using a descriptive study, we collected data via a literature review, focus groups, and an expert panel to inform the scale domains and blueprint design. Pretesting (cognitive interviews) was used to inform item revision decisions. An item analysis was also conducted for the responses provided in the cognitive interviews. Item statistics (means and SDs), interitem correlations, and reliability were examined. Data were analyzed using SPSS (IBM Corp) software.

**Results:**

A valid and reliable 42-item HPV vaccine communication competency scale was developed. The scale included 6 domains of interest. Scale items were moderately to strongly correlated with one another, and Cronbach α indicated good internal consistency with each scale. Scale items included were related to provider introduction or rapport (α=.796), patient respect or empathy (α=.737), provider interview or intake (α=.9), patient counseling or education (α=.935), provider communication closure (α=.896), and provider knowledge (α=.824).

**Conclusions:**

Pharmacists, nurse practitioners, and physician assistants should be trained to be competent in HPV vaccine communication and recommendation due to their expanded roles. Interdisciplinary collaboration is important to account for the trainee’s individual differences and ensure the best health care outcomes for patients. A standardized HPV communication scale can be used to ensure effective and consistent recommendation by health care providers, thus affecting immunization rates.

## Introduction

### Background

An increased demand for nurse practitioners and physician assistants as primary care providers has been observed in the last 10 years in the United States. This was primarily initiated because of population growth, the aging population living longer, and health insurance expansion [1]. However, primary care providers are also pivotal to immunization administration and uptake. Apart from the traditional clinical setting, we have seen an increase in the number of nurse practitioners and physician assistants working in settings such as urgent care clinics, convenient care clinics, and retail clinic service provider sites such as The Minute Clinic [1,2]. This is a great advancement in trying to bridge the gap in the health care (specifically immunizations) industry particularly as it relates to accessibility, cost, and convenience. However, the scope of practice (full, reduced, or restricted practice) is determined by the state legislative and regulatory barriers [1]. In the state of Georgia, a nurse practitioner’s and physician assistant’s practice and prescription authority are overseen by the supervising physician and possible written protocol enacted by that physician [3].

Apart from nurse practitioners and physician assistants, pharmacists have also been identified as critical to immunization uptake among adolescents [4]. As the COVID-19 pandemic has affected medical visits and school-based interactions because of social distancing, stay at home orders, and governmental bans, we have observed that the number of vaccinations, including the vaccination for human papillomavirus (HPV), declined dramatically over a short period especially among children and adolescents [5]. Coupled with the already lagging vaccination rates in some states before the COVID-19 pandemic, this is cause for concern [6]. It is estimated that since March 2020, ordering and billing for the HPV vaccine have dropped by almost 20% with administration rates remaining down between 20% and 40% in June 2020 [7]. Before the pandemic, Georgia had stricter laws prohibiting a pharmacist’s ability to administer immunizations. However, pharmacists are now able to provide HPV vaccination in addition to other routine vaccines [8]. Therefore, pharmacists, nurse practitioners, and physician assistants must be competent concerning HPV vaccine recommendations and patient communications to help bridge the gap.

As curriculum competencies for HPV vaccination information and communication strategies vary within each program, including other programs such as family medicine, there is a need for a standardized communication scale [9]. Although there is a Medical Communication Competency Scale [10,11], this scale is primarily focused on general medical communication during medical interviews and does not tailor scale items to measure the effectiveness of communication language and skill set necessary for HPV vaccination uptake. Therefore, an HPV vaccination communication scale must be developed. This is particularly important when interacting with specific communities, community groups, and disparate population groups as they are most affected by HPV-related conditions and diseases. Research literature indicated that health care providers and parents were more accepting of females being vaccinated for HPV than males, which limited researchers’ understanding of HPV-associated diseases among men [12]. According to McGhee et al [13], there is limited understanding of HPV-associated diseases among men, which may be related to low acceptance of HPV vaccinations in males. In addition, HPV incidence disproportionately affects minority racial and ethnic groups. Black women are less likely to complete the series of vaccines compared with White women [14]. Black and Hispanic individuals are more likely to be affected by HPV-related morbidity and mortality rates compared with White individuals [15]. This is a direct result of missed clinical opportunities and a lack of proper recommendations during patient visits with health care providers, which is possibly because of provider discomfort [13,16]. Due to this, the Centers for Disease Control and Prevention cancer panel encourages efforts to improve comprehensive communication strategies for primary care providers and other health care professionals [16].

### Clerkship and Residential Training

Although the enrollment in medical, physician assistant, nurse practitioner, and pharmacy programs have increased, we are witnessing shortages of clinical education through clerkship training in the United States. Clerkships (clinical rotations) are immersive learning opportunities where students are provided real-world opportunities to apply their understanding of clinical and scientific concepts to patient care during their rotations. They are supervised and observed by senior faculty and take a primary role in obtaining information and developing final treatment plans [17].

Shortages are even further compounded by a decrease of preceptors, hospital mergers, or health system closures [18]. It is expected that during the clinical phase of education, a medical trainee puts into practice what they have learned. Furthermore, it requires clinical preceptors to supervise students in their performance of routine tasks for optional and fundamental learning [19]. The structure of medical and clinical education relies on the premise that supervisors possess the competencies needed to guide and assist in the training of those under their direction. It also relies on the premise that medical trainees are competent to operate as a health care provider or primary care provider. Using the newly proposed HPV communication competency scale in clinical education training before real-world experiences, medical trainees would have experiential learning opportunities that would improve long-term health for their future patients. For primary care providers already practicing in health care, the use of the HPV communication competency scale with the addition of simulation-based training (modules providing practice examples of effective patient-provider communication) would provide safe spaces to improve upon skills needed to promote HPV vaccination. It could also highlight the need for enrollment in up-to-date continuing education training and curriculum programs, as well as effective system-wide assessments. This would ensure that from a health care system perspective, providers can receive the level of clinical education needed to meet performance, communication, and recommendation standards.

### Communication Training in Medical Programs

Finally, although the demand to fill the immunization health care gaps is increasing, there is no way currently to assess the level and extent of communication training and preparation that currently exists in programs throughout the United States surrounding HPV vaccine uptake. However, there is an opportunity to develop a communication scale measure with common standards to be used as an assessment tool among medical trainees surrounding HPV vaccination. This assessment can be implemented at key points in the curriculum of students in nurse practitioner, physician assistant, and pharmacy programs to ensure that graduating students are fully competent to provide effective recommendations and are comfortable with the information that should be conveyed, thereby resulting in parents and patients who can make informed decisions related to HPV vaccine uptake.

### Literature Review

Physician and medical practitioner recommendations are a key predictor of HPV vaccination. However, the literature continually cites missed opportunities by providers leading to low vaccination rates. According to the Henry J Kaiser Foundation, >1 in 10 parents of adolescent girls and 1 in 5 parents of adolescent boys said the vaccine was not recommended to them by health care providers [20]. In another research study, 23 focus group sessions (n=112) were conducted with women (aged 18-26 years), parents, community leaders, and health care providers in Ohio Appalachia. During these sessions, it was found that health care providers “were the only type of group that did not mention the importance of explaining how vaccines work or the pros and cons of HPV vaccinations a part of educational programs” [21]. In a national survey of 1400 respondents conducted by the National Cancer Institute, it was found that only 1 in 4 youths talks to a health care provider about the HPV vaccine. In the same study, it was reported that when asked about vaccine efficacy, 70% of the providers did not know how effective the vaccine was [22]. This is primarily because of (1) their lack of knowledge about HPV and the manifestations of the disease, (2) being more likely to discuss HPV with patients if they had a positive HPV diagnosis, and (3) their reluctance to talk to their young patients about sex [23-25]. In a qualitative study on physician HPV vaccination practices, it was found that physicians (1) did not feel it was their role to provide HPV vaccination and that auxiliary health care service agencies such as the health department should be charged with that responsibility, (2) stated the need for more information about the safety and efficacy of the vaccine before they could recommend and administer it, and (3) were critical of the policy recommendations for the HPV vaccines [24].

When we look at key knowledge of the HPV vaccine, only 35% of health care providers who participated in large-scale US surveys were able to correctly identify that most genital HPV infections resolve without any treatment. In addition, only 47% of the health care providers knew that the HPV strains associated with genital warts differ from strains usually associated with cervical cancer and only 63% of the health care providers believed that HPV infection increases the risk of anogenital cancer in men [26,27]. This lack of information can partly be because of the ever-changing narrative surrounding the HPV vaccine. As the vaccine was first recommended in early 2009 for use, we have seen several changes and adjustments in the last 10 years including several iterations of the vaccine (Cervarix, Gardasil 4, and Gardasil 9 at present), the shift from only female focus to include males, catch up vaccination groups, the emphasis for vaccination among the age group of 9 to 12 years for best immune response, and now new recommendations for 2 versus 3 dose series dependent on the age of initiation [28]. Due to this, many health care providers including primary care providers have been cited in the research as having limited understanding, evident knowledge gaps, uncomfortableness, and even confusion on how to recommend the vaccine to their young patients and parents [27,29,30]. Therefore, it has been recommended that patient-provider communication incorporates a strong endorsement with an emphasis on cancer prevention and same-day vaccination to improve the lagging rates [31]. Therefore, the proposed project will not only add to the research literature and thus increase the body of knowledge surrounding HPV patient-provider communication but also create a practical tool that can be used to improve health care and public health outcomes overall. Therefore, the specific aim of this research study was to develop a reliable and valid HPV vaccine communication scale that can be used to measure the competency of primary care providers when recommending the need for vaccination to parents and patients. Specific research questions included were as follows:

1. What factors related to provider communication comfort should be considered to increase the effectiveness of HPV vaccine recommendations when given?
2. What HPV vaccine communication competencies are relevant to the nurse practitioner, physician assistant, and pharmacy professions?

## Methods

### Overview

**Figure 1:**
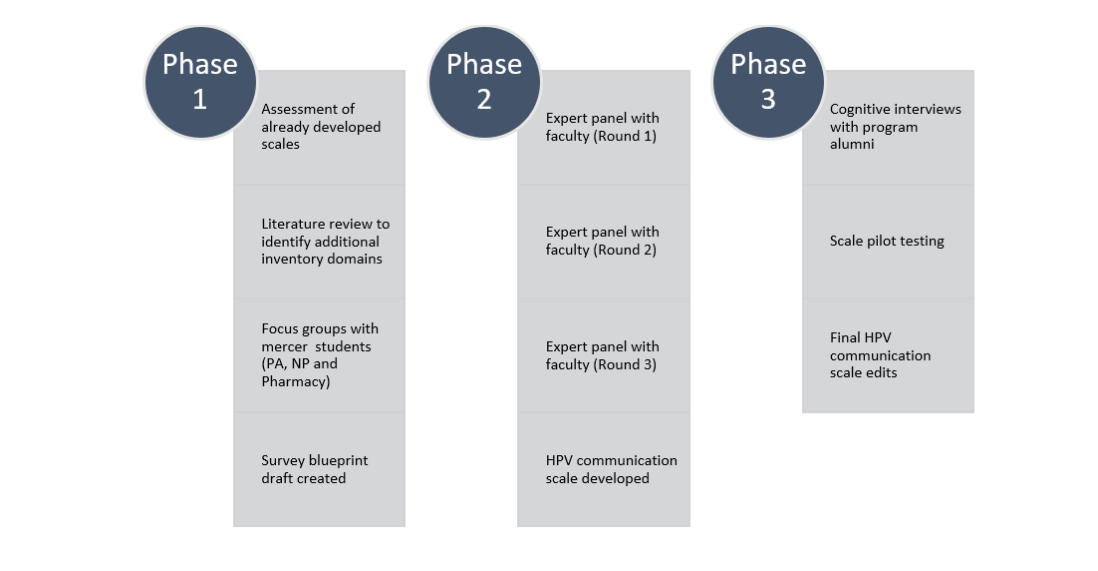
Methodology schema. HPV: human papillomavirus; NP: nurse practitioner; PA: physician assistant.

Scale domains were developed using a variety of different approaches identified below (Figure 1). Previously developed scales and HPV literature were reviewed to identify scale initial domains. Focus groups were facilitated to identify perspectives related to HPV vaccine communication that will be incorporated in the development of scale items. An expert panel was conducted to provide insight on scale content, word choice, and appropriateness, and finally, cognitive interviews were conducted with practicing professionals to assess the thought process involved with responding to the scale items and pretest scale items [32-35].

To mitigate the risk of contracting COVID-19, the project methodology was designed to collect data for all phases using web-based platforms or technology. This ensured that the risk of COVID-19 exposure to the investigators, staff, research participants, and extended networks was minimal to none.

### Phase 1—Conceptualization and Domain Specification

We conducted a literature review to identify existing validated measures and concepts related to health communication, HPV, and HPV recommendation to begin our research. Although some scales relevant to medical communication and communication skill assessment exist, none of them were specific for HPV and HPV vaccination. Therefore, while the literature review data collection process was being conducted, focus group sessions were also held to capture data from current medical trainees in Mercer University programs. The focus group sessions were used as a complementary data collection inquiry to fill in the gaps relevant to additional scale domains. As focus groups are a frequently used qualitative approach to gain an in-depth understanding of issues, it was determined appropriate for this study [36].

Focus group participants were recruited using convenience sampling. Clinical and medical directors of all programs were asked to distribute a recruitment email to students for participation in the study. Using SuperSaaS web-based scheduling software, students were able to self-enroll in the focus group session that worked best for their schedule [37]^.^ Following confirmation of enrollment, a web-based informed consent form was emailed to the participant encouraging completion before participation by the lead principal investigator. Qualtrics (Qualtrics XM) was used to collect informed consent and basic participant information. All focus group sessions were facilitated over the Zoom videoconferencing platform.

In February 2021, the team facilitated its first focus group session with students in the nurse practitioner or nursing, physician assistant, and pharmacy programs. On the basis of the feedback provided, the focus group protocol was updated, expanded upon, and resubmitted to the institutional review board for approval. This was done because some questions needed to be reworded or updated for additional clarity. In March and April 2021, additional focus group sessions were facilitated. The average size for the focus group sessions was 8 persons. Sample size per group was 9 for the first group, 8 for the second group, 7 for the third group, and 9 for the fourth group. Key topics explored during the focus group sessions included curriculum exposure to HPV vaccine or virus information, current HPV vaccination recommendations for adolescents, recommendation competency and comfort, additional training in HPV, health provider communication, communication tools to improve HPV vaccine recommendations, and vaccination strategy. The entire focus group protocol questionnaire can be viewed in Multimedia Appendix 1.

All focus group sessions were recorded and transcribed for further analysis by our graduate research assistant. Our graduate research assistant also served as a notetaker, so no information was missing. All the qualitative data collected from the focus group sessions were coded using NVivo (QSR International) software. A codebook was established deductively using the interview script as a baseline. Additional codes were added, defined, and expanded upon as the coding process continued using the constant comparative method based in grounded theory. To establish interrater reliability of emergent themes, the researchers developed a schedule for coding. Each week, the researchers would code 10% of the transcripts independently and then merge coding findings into NVivo to run a coding analysis. The research team would then meet to discuss the codes identified until 90% to 100% agreement was achieved for the data set. Differences were also discussed throughout the data analysis until a consensus on the coding scheme was established.

Following the completion of the literature review and focus group sessions, a blueprint of a scale was drafted. Included components were derived from 10 articles or scales of interest [10,38-46]. Feedback and data collected from the focus group sessions were also integrated into the initial blueprint of the scale. The blueprint of the scale was then provided to the research team for individual feedback. Once all individual feedback was combined into a master document, the principal investigator and coinvestigators collectively assessed specific domains and agreed upon the final draft of the questionnaire to be used in the next phase of the research study. In total, 25 domains were derived from existing instruments or concepts from the research literature and 20 domains were newly developed or adapted from discussions from focus group participants given a total number of 45 items.

### Phase 2—Expert Review and Item Development

We established a panel of expert faculty at Mercer University trained in the fields of nurse practitioner, physician assistant, and pharmaceutical studies in July 2021. The principal investigators identified 10 potential members based on their experience with training medical trainees, communication expertise, and immunization practice. Before their enrollment in the Delphi panel, the scale was entered into Qualtrics. Items were cross rated within Qualtrics by each expert panelist relevant to the domains of interest: *provider introduction or rapport, patient respect or empathy, provider interview or intake, patient counseling or education, provider communication closure, and provider knowledge*. Each expert panelist was provided 1 week to review the scale items in Qualtrics and provide feedback on each section. The assessments of the domains were done individually and free from the influence of other members. The feedback was then compiled by the research team, discussed, agreed upon, and then implemented for subsequent rounds. In total, 3 rounds of feedback were conducted, and items were evaluated within Qualtrics by the expert panel for relevance and appropriateness (1 round) and clarity (2 rounds).

A fourth round was implemented to collect preliminary data concerning the scale’s reliability and validity. To facilitate this, a digital stimulus (1 minute and 40 seconds video) was provided to the expert panel, and they were asked to evaluate the provider’s HPV vaccine recommendation as viewed in the video. An item analysis was conducted for the purpose of determining the quality of the items. Item analysis included item statistics (means and SDs), interitem correlations, and reliability. SPSS software was used to conduct data analysis. As this version of the scale included not applicable as a response item, response option differences emerged between the disciplines regarding their evaluation of the providers competence. This led to some scale items not being well correlated to one another. However, for all domains of interest, Cronbach α values were reported as between .7 and .9. Following the completion of the Delphi panel, the research team met again to review the scale items with particular interest in the items not well correlated. Upon the review of feedback provided by expert panelists, the data itself and recommendations from our research and evaluation methodologist items were (1) updated, (2) reorganized, or (3) removed from the scale. After the Delphi Panel, a total of 43 scale items remained.

### Phase 3—Cognitive Testing

A completed list of items was then finalized to be examined during the cognitive interview phase of our research study. An additional graduate research assistant was hired who assisted with the enrollment of study participants using SuperSaaS software [37]. Convenience sampling was conducted with alumni of Mercer University’s nurse practitioner, physician assistant, and pharmaceutical studies programs. However, due to competing COVID-19 pandemic response demands on practitioner enrollment and study attrition, the recruitment strategy was updated to include all available practicing practitioners that were interested in the study. Strategies implemented to recruit the latter group of practitioners included emailing national and state professional organizations relevant to the disciplines. Cognitive interviews were facilitated to garner additional feedback regarding the clarity and appropriateness of the scale items. Once scheduled, the first round of interviews was conducted by both graduate research assistants using Zoom web-based videoconferencing software. Transcripts were auto-produced and then cleaned before being analyzed using NVivo software. Notes taken during the cognitive interviews were also compiled and assessed by the research team. Final edits to the scale were made before round 2 of the cognitive interviews.

Similar to the fourth round of the Delphi panel, a second round of the cognitive interview was implemented to collect preliminary reliability measures and conduct a correlation analysis between scale items. To facilitate this, a digital stimulus (1-minute-and-40-second video) was provided to the round one cognitive interview participants, and they were asked to evaluate the provider’s HPV vaccine recommendation as viewed in the video. To address issues with correlation identified previously, the not applicable (N/A) response item was completely removed from the scale, and a question asking respondents to identify their discipline was added. An item analysis was also conducted for the responses provided in the cognitive interviews. Item statistics (means and SDs), interitem correlations, and reliability were examined. Data was analyzed using SPSS software. Overall, scale items and subscales were well correlated with one another. However, there was 1 section where statements were too highly correlated with one another (redundant in nature). After further analysis and investigation, 1 statement was removed.

### Ethics Approval

This study was approved by the Institutional Review Board for Human Subjects Research at Mercer University in accordance with the 2018 Federal Regulations 21 CFR 56.110(b) and 45 CFR 46.110(b) (for expedited review) and was approved under categories _6, _7 per 63 FR 60364 in November 2019 (approval number: H1911287).

## Results

### Focus Group Sessions

A total of 4 focus group sessions were conducted. This phase sought to seek insight from a targeted 40 students; however, only 33 of them participated. Demographic breakdown of the participants was as follows: nurse practitioner (20/33, 61%), physician assistant (9/33, 27%), and pharmacy (4/33, 12%). Emergent themes that arose from the focus groups were focused on trainees’ comfort with effectively communicating HPV and HPV vaccine information to all patients (Textbox 1).

**Emergent themes and subthemes related to medical trainee human papillomavirus (HPV) vaccine experiences and competence (focus groups).**

**Themes and subthemes**

- Additional insight: participants shared statements related to additional insight or ideas relating to scale development
- Additional training opportunities: statements related to ideas on ways to prepare medical trainees to be comfortable and competent in their ability to recommend the HPV vaccine
- Comfort: participants’ self-rated comfort level as a health care provider in recommending the HPV vaccine to current and future patients on a Likert-like scale from 1 to 10
- Comfort scale development: statements related to the development of a scale to measure the comfort level of health care providers who recommend or communicate the need for HPV vaccination
- Competence: participants’ self-rated competency as a health care provider in recommending the HPV vaccine to current and future patients on a Likert-like scale from 1 to 10
- Competence scale development: statements related to the development of a scale to measure the competence of health care providers who recommend or communicate the need for HPV vaccination
- Current HPV knowledge: the demonstration of HPV knowledge including general information about virus and vaccine. As well as including information about current vaccine recommendations
- HPV: participant knowledge of HPV
- HPV vaccine: participant’s knowledge on HPV vaccine
- Human papillomavirus: participant’s knowledge on the human papillomavirus
- Health care provider HPV communication: statements related to what the participant’s believe health care providers should discuss and communicate about HPV vaccination
- Additional communication tools: statements related to additional tools or methods health care providers can use to communicate with patients regarding the HPV vaccine
- Knowledge exposure: statements relating to participant’s exposure to information on HPV as well the vaccine
- Class or opportunity: setting in which participants were exposed to HPV information or experience
- Depth: level of exposure to HPV information
- Profession: chosen field of study or work, including reasons why the profession was chosen
- Vaccination completion: statements about methods a health care provider can use to ensure vaccination initiation and completion

For example, when discussing their comfort level, 1 participant shared that bringing up HPV and the vaccine with parents may feel taboo because “nobody wants to think that their 12-year-old is having sex.” Participants indicated that their lack of competency is because of a deficiency in knowledge regarding certain aspects of HPV and HPV vaccination as seen in the following quotes:

Like I think I would struggle with the most of that, and then I probably would need to do a little bit more research on protocols and guidelines, as you know, like when the HPV vaccine like should really be given and the doses, and things like that.

I’ll be able to educate them about the purpose of the actual vaccine, the signs and symptoms. To look for what [is] HPV, how [it] is transmitted, prevention methods, but in terms of like the frequency of the vaccination I’m not quite sure and, like the age cutoff, I’m not too familiar with what I do know what the adverse outcome is if you have HPV.

The participants also discussed the differences in competency between medical professionals concerning HPV and the HPV vaccine. Nursing and physician assistant students who saw and interacted with adolescents in primary care settings felt more competent in their ability to give a strong high-quality HPV vaccine recommendation that compared with their peers who do not work in that setting or disciplines such as pharmacy. This is exemplified in the following quotes:

I think the difference in like pharmacy and nursing is [that] I would be able to talk more about the disease than I probably could [on the] vaccine itself, whereas pharmacy would probably know all about the vaccine.

I could definitely do like the risk and the benefits of it, but as far as like the details of the guidelines, I would definitely need to go and like update myself and like just educate myself a little bit more before I went in and tried to give somebody like the rundown on what that is.

I also haven’t had any experience giving vaccines, but I have had a lot of experience talking with patients and deciding treatment plans and everything like that.

I’ve not been exposed to an opportunity to give it. I don’t deal with children, you know. And I’m dealing with primarily pregnant women, anyway, so we’re well beyond all that, so I just don’t have any exposure to like giving lots of vaccines anyways.

The quotes further emphasize the importance of developing a scale to assess medical trainees on their ability to recommend the HPV vaccine. The focus group sessions also provided vital information on the differences in HPV and HPV vaccine education and training among medical professionals. This scale could provide the opportunity for medical trainees to be assessed and increase their comfort and competence, allowing them to appropriately bridge the gap between science and fiction as they move forward in their careers as health professionals.

### Cognitive Interviews

In total, research sought to complete 33 sessions but only 15 participants completed both rounds of the cognitive interviews. Emergent themes and subthemes from this phase of our data collection are included in Textbox 2.

**Emergent themes and subthemes related to human papillomavirus (HPV) Vaccination Communication Scale clarity and relevance (cognitive interview sessions).**

**Themes and description**

- Scale: a graduated range of values forming a standard system for measuring or grading something. Including statements relating to scale measurements, that is, novice-expert
- Generalizability: statements related to the applicability of the HPV Recommendation Competency Scale across multiple disciplines
- Scale item: statements about individual scale items including statements related to the necessity, appropriateness, and flow of the items
- Assessment potential: individual describes either ease or difficulty in using scale item to assess student providers, including feasibility
- Clarity: statements that relate to the participant’s understanding of the scale item
- Confusion: participants indicate confusion with scale item or wording
- Rephrase: participants suggest rephrasing or changing words in items
- Positive comments: positive statements about scale items including wording, flow, and appropriateness. Can include statements of agreement regarding scale items.
- Redundancy: statements indicating either items or sections as being repetitive
- Scale section: statements related to any of the 6 scale sections, including overall flow and appropriateness

Upon the completion of this phase of the study, we were able to run reliability and validity tests. Cronbach α values for each section were as follows:

- The provider introduction or rapport subscale included 7 items (α=.796).
- The patient respect or empathy subscale included 4 items (α=.737).
- The provider interview or intake subscale included 6 items (α=.9).
- The patient counseling or education subscale included 10 items (α=.935).
- The provider communication closure subscale included 7 items (α=.896).
- The provider knowledge subscale included 8 items (α=.824).

## Discussion

### Principal Findings

HPV, a sexually transmitted infection, is a highly prevalent disease [47,48]. Approximately 79 million Americans are currently infected with HPV, with 14 million becoming newly infected annually [47]. To help prevent the increasing rate of cancers associated with HPV, the HPV vaccine (Gardasil 9) was developed to protect against persistent infections of associated HPV subtypes [49,50]. Despite having access to licensed HPV vaccines for more than a decade, HPV vaccination rates are still lagging among many adolescents and the coverage varies in the United States. According to Sonawane et al [51], nearly 46% of adolescents who are eligible are not up to date with the recommended HPV vaccination as of 2019. According to the National Immunization Survey 2008 to 2018 teen data, although vaccination initiation rates have increased over the last years, completion and up-to-date rates still fall below 50% in most US states [52].

Although there are many barriers to HPV vaccination, parental vaccine hesitancy has been one of the most constant factors affecting vaccination rates among the adolescents [51]. To combat this, it has been recommended that providers have strong high-quality recommendations. Current research has shown that adolescents who report receiving a strong high-quality recommendation from their provider are >8 times more likely to initiate and completed HPV vaccination than those who do not [53]. However, many providers and trainees do not feel as though they possess the competence to provide a strong high-quality recommendation. This is not only evident in the previous research but also in the results obtained in this study. To increase communication comfort, study participants indicated that they needed to receive more information or knowledge about the HPV and vaccine guidelines especially if they were not working in a clinical setting where they would interact with them on a consistent basis. Nurse practitioner and physician assistant trainees communicated that they felt more confident in their ability, when compared with pharmacy trainees who are a new addition to the implementation of HPV immunization programs. It was evident that professional protocols concerning patient interactions, communication standards, and even training differed among the disciplines. This is further compounded by state variation concerning the scope of practice to include vaccine age—eligible adolescents (independent authority, collaborative practice agreement, or by prescription only) [54]. However, with the changes to pharmacy immunization protocols owing to the COVID-19 pandemic challenges, there is increasing support by primary care physicians and parents alike for trained pharmacist HPV vaccination administration as an independent authority [54,55].

Our study also sought to identify what specific communication competencies were needed relevant to the 3 disciplines of focus (nurse practitioner, physician assistant, and pharmacy). Although there were key differences that emerged, the 3 phases involved in this research study concluded that the communication process was an interactive and yet integral role of each clinician. Upon information collected from the literature, and feedback gathered by our focus groups and Delphi panel of experts and practicing clinicians, and validation through our cognitive interviews, it was identified that (1) provider introduction or rapport, (2) patient respect or empathy, (3) provider interview or intake, (4) patient counseling or education, (5) provider communication closure, and (6) provider knowledge were important components during a patient-provider interaction. The subscales identified and confirmed throughout our study match the ones identified in similar research studies that focused on enhanced HPV communication tools for vaccine hesitant parents. As our scale statements assess a medical trainee’s competency related to presumptive communication and motivational interviewing techniques it can be more effective in alleviating parental vaccine hesitancy [56]. As a strategy to also address the diverse population subgroups served, the scale questions included provide the opportunity for tailoring recommendations and vaccine communication based on race and ethnicity, gender, sexual identity, and biology differences, to name a few. While this may not close the gap completely, as the scale was developed through a health equity lens, it can serve as a tool that moves the needle forward concerning improvements in HPV vaccination disparities.

Despite the successes of this study, there were some limitations. First, the curriculum development and training of nurse practitioners, physician assistants, and pharmacy trainees varies heavily, and as such, exposure to HPV and HPV vaccination information is not consistent and equally weighed. Therefore, although these 3 practitioners have been integral in addressing the lag in medical care provisions, specifically HPV vaccination, the differences among professions may also influence how and in which ways the scale can practically be used. Second, it requires that supplemental training be developed and tailored to each individual clinical program’s curriculum to ensure standardization and effectiveness of the scale tool when used in the field. Third, although health care providers and professionals are integral to the success of vaccination initiatives and programs, they are not the only stakeholder involved. Other important adolescent reference groups for decision-making may also involve parents, teachers, or peers. However, due to the scope of this study, and the intentionality of addressing the pressing needs for high-quality provider communication as cited heavily throughout existing research, they were not included as participants in this study.

### Future Implications

Although there has been much effort toward making HPV recommendation communication standardized through the publications of clinician action guides (physician, physician assistants, nurse practitioners, nurses, medical assistants, and dentist or dental care providers) by the National HPV Vaccination Roundtable, more efforts need to be made concerning the development of standardized education and training modules for each profession involved in HPV vaccination [57]. Since research has shown that strong provider recommendation is highly associated with increased vaccine initiation, completion, follow-through, and decreased parental hesitancy, it is important that training is broad enough yet specific to ensure that no matter the profession, all participating practitioners possess the communication skill sets needed to provide the strong and high-quality recommendation needed [58]. This is even more important now, as we see key disparities among the HPV-related cancers in the United States. Additional work should also explore the perspectives of key stakeholders such as parents, peers, and teachers on HPV vaccine decision-making for initiation and completion, with specific understanding toward health equity and application toward multiple priority populations.

### Conclusions

Immunization against preventable cancers associated with the HPV has been a top priority for more than a decade. With the introduction of Cervarix, Gardasil 4, and now Gardasil 9 vaccines, greater importance has been placed on vaccinating adolescent girls and boys starting at the age of 9 years for achieving the best immune response and vaccine efficacy. However, there have been many barriers for HPV vaccination, with the most influential barriers prohibiting initiation and completion being parental resistance and refusal [59]. To combat this, the Centers for Disease Control and Prevention, Advisory Committee on Immunization Practices, President’s Cancer Council, and American Academy of Pediatrics have all emphasized the need for strong high-quality recommendations by health care providers [60]. However, since there is no standardized training program on HPV and HPV vaccine education, and major differences exist among the nursing, physician assistant, and pharmacy professions, the development of a reliable and valid HPV vaccine communication scale is important for increasing vaccination rates. Since this study collected data using an interdisciplinary approach, we believe that the communication scale developed accounts for communication needs and training differences among health care professions. Using the scale alongside a training module such as the *HPV Vaccine: Same Way Same Day* smartphone app will ensure that medical trainees and current practicing professionals can maintain the competence needed to provide high-quality HPV vaccine recommendations regardless of the setting, improving immunization rates and reducing future HPV-related cancer trends [61-63].

## Appendix

Multimedia Appendix 1 Focus group facilitator protocol.

## Abbreviations

HPV: human papillomavirus
