# Supplementary material for: HPV Vaccine Communication Competency Scale for Medical Trainees: Interdisciplinary Development Study
Source: JMIR Form Res. 2022 Nov 4;6(11):e38164. doi: 10.2196/38164 (PMC9675010; doi:10.2196/38164)
Supplement: Multimedia Appendix 1 [file formative_v6i11e38164_app1.pdf]

FOCUS GROUP PROTOCOL

Moderator: *Thank you so much for coming here today. My name is ______________and I am here to facilitate this focus group. The reason you were invited to participate in this focus group session is because we want to collect information on what all should be included in a scale to measure HPV vaccine communication competence and comfort level among healthcare providers. We would like learn from conversations with a few of you about your own ideas on how this should be done. This session will last approximately 60 minutes and will audio recorded so that no bit of information is missed. ____________, our Research Assistant will also be note taking during this session to capture your ideas. Audio recordings will not be heard by anybody other than the research team and your responses will be kept confidential. The findings will only be released in the form of general topics with no identifiable personal information. Do I have your permission to record our discussion?*

(After permission is granted moderator will move on to focus group questions).

1. **Icebreaker** – If you could practice (get a job) anywhere in the world, where would you go and why?
2. What made you decide to go into your chosen profession?
3. Thinking about your program, in what ways you been exposed to information related to the HPV vaccine and HPV virus?
   a. Describe the class/ opportunity that exposed you to this information and the semester/year it occurred.
   b. How extensive was the information provided?
   c. What do you know about the HPV virus/ HPV vaccine?
   d. Describe what you know about current recommendations for HPV vaccination among adolescents.

Moderator Prompt: *The next set of questions, will ask you about your comfort and competence as a healthcare provider. For the purposes of this study, competent can be seen as being well informed and knowledgeable about the facts related to HPV and the HPV vaccine. On the other hand, comfort is associated with having opportunities to practice giving vaccine recommendations while navigating situations of noncompliance or vaccine hesitancy.*

4. Using a scale of 1 – 10 where 1 represents not at all competent and 10 represents very competent, how would you rank your competence level regarding recommending the HPV vaccine to future patients and parents? Why did you give yourself that score?
   a. **Follow-up**: In developing a scale to measure the competence of healthcare providers who recommend/ communicate the need for HPV vaccination, what should be included?

5. Using a scale of 1 – 10 where 1 represents not at all comfortable and 10 represents very comfortable, how would you rank your comfort level regarding recommending the HPV vaccine to future patients and parents? Why did you give yourself that score?
    a. **Follow-up:** In developing a scale to measure the comfort level of healthcare providers who recommend / communicate the need for HPV vaccination, what should be included?
6. Besides what you have learned in your program thus far, what else should be used to prepare medical trainees to become comfortable and competent in their ability to recommend the HPV vaccine?
7. When recommending the HPV vaccination, what all should healthcare providers discuss or communicate?
    a. **Follow up:** Are there any additional communication tools, a healthcare provider can use to increase the effectiveness of their recommendation?
8. How can you ensure that your patients not only initiate or start the vaccination series, but come back to get the 2nd and 3rd shots?
9. Is there anything else that you would like to add that could be of further insight or benefit to the scale development?

Closing: Thank you for taking the time out of your busy schedules to participate in this focus group session. All feedback that was provided will help us greatly. We hope you have a great remainder of your day!

**language change to past tense for alumni focus groups.*
